# Supplementary material for: The use of laser lead extraction sheath in the presence of supra-cardiac occlusion of the central veins for cardiac implantable electronic device lead upgrade or revision
Source: PLoS One. 2021 May 14;16(5):e0251829. doi: 10.1371/journal.pone.0251829 (PMC8121537; doi:10.1371/journal.pone.0251829)
Supplement: Attachment — Submitted filename: Response to Reviewers.docx [file pone.0251829.s002.docx]

Rebuttal Plos one Journal

Dear Sir or Madam,

We are pleased to resub mit a major revision of the enclosed manuscript entitled: “The use of Laser lead extraction sheath in the presence of supra-cardiac occlusion of the central veins for cardiac implantable electronic device lead upgrade or revision.

” for publication in the Journal of Cardiothoracic Surgery.

We would like to express our appreciation for giving us the opportunity and the motivation to improve this paper with very valuable suggestions and elaborate comments.

The manuscript underwent substantial revision. Details of each change to the original manuscript are reported below along with each issue raised by the Reviewers. In the manuscript, modified text passages are marked in red.

We thank you for helping us to substantially improve this paper.

Sameer Al-Maisary and Co-Authors

Comments from Reviewer #1:

The implantation of cardiac implantable electronic devices (CIED) has very increased in the last decades, reducing mortality and improvement the quality of life of patients with cardiac rhythm disorders. In this scenario the use of laser-assisted lead extraction (LLE) is a is a very useful technique, particularly in the presence of bilateral subclavian, innominate or superior vena cava obstruction, that is a major limitation to device revision and/or upgrade.

I would like congratulate all the authors contributing to this good article and research work. The article is presented in an intelligible fashion.

In conclusion, given the overall work, I recommend minor revision concerning a implementation of bibliography, reporting for example the group of Prof Curnis A, who direct a center of great experience in this field (see the paper "Curnis A, Bontempi L, et al. Active-fixation coronary sinus pacing lead extraction: a hybrid approach. Int J Cardiol. 2012 May 3;156(3):e51-2. doi: 10.1016/j.ijcard.2011.08.016").

Response:

We thankfully acknowledge the compliments of Reviewer 1.

We read the work of Curnis et al and found it interesting. We added it to our conclusion section to mark the complexity of some Lead extractions. We thank you again for suggesting this reference to enrich our paper.

Comments from Reviewer #2:

NO COMMENT TO ADD. IT'S A GOOD REVIEW OF PATEINTS FOR A CENTER. I CAN ADD THAT IN SAME CASESE IS USEFUL TO HAVE A WIRE FROM FEMORAL VEIN TO RIGHT ATRIUM, IN CASE TOU NEED IN EMERGENCY TO INSERT A BALOON

Response:

Thank you for your comments. You have raised an important point here. We agree that inserting a wire from the femoral vein is helpful but because of the cost of the balloon we are not using it. We try to get enough financial support to be able to use it but for the time being we cannot afford it.

Comments from Reviewer #3:

 I have read with interest the paper entitled “The use of Laser lead extraction sheath in the presence of supra-cardiac occlusion of the central veins for cardiac implantable electronic device lead upgrade or revision.”
The paper is a retrospective analysis of patients who underwent transvenous lead extraction (TLE) requiring Laser. The Authors conclude that this approach has good results in terms of safety and efficacy.
Some specific comments are:

-The section “conclusion” in the abstract is a repetition of the results and not a real conclusion. Please add that data in the section results and rearrange the section conclusion with correct form.

Answer:

We thank the reviewer for pointing this out. We have revised the conclusion completely.

-It is not well described the method used for demonstration of vein occlusion (venography? CT scan, other?). Please better explain this in the method section and in the results section (how many patients performed a unilateral or bilateral venography? A CT scan?).

Answer:

This observation is correct. We have changed the sentences and made it more clear that Venography was used to make the diagnosis of venous obstruction in 23 patients. We made it also more clear in the methods section, that Venography was used for the diagnosis. We also added the word bilateral to venography as all patient with obstruction of an access vein undergo venography of the contralateral side.

- It could be of interest to know how many leads had an active or a passive fixation system.

Answer:

We thank the reviewer for pointing this out. We are sorry to tell you, that we don’t have recording of the LV-Lead type (active or passive)

- It could be of interest the median duration of the follow up

Answer:

We appreciate the reviewer’s insightful suggestion and agree that it would be useful to demonstrate that. However, trying to get these data again well need a lot of time and mostly we want be able to get the whole data. Because of that we concentrated on the first 30 days.

- line 229 (discussion): you say that Lead laser extraction has shown good results. In proof of this, you report the results of the ELECTRA. It is misunderstanding, because you do not explain that only a minority of patients of the ELECTRA underwent Laser extraction (19.3%). Additionally, a sub analysis of the ELECTRA revealed that the use of mechanical dilatation was a predictor of lower incidence of major complications, compared with powered sheaths. I think that you have to explain better data from ELECTRA and extend the discussion also with an analysis of the other non-laser approach.

Answer:

We thank the reviewer for pointing this out. We have revised our discussion and adapted your suggestions. We added that 19.3 % of the patients underwent laser lead extraction. We also added the Wazni et al paper to support the benefits of laser lead extraction to the discussion. We also discussed the role of the mechanical extraction and that we still have to improve it, to avoid the risks of laser lead extraction.

-In conclusion, the topic is of interest, and the paper could be suitable for the publication with the correction of these major revisions.

Answer:

Thank you for your assessment. We hope that through our correction, we reach your expectations.

Once again, we thank you for the time you put in reviewing our paper and look forward to meeting your expectations. Your inputs have been precious. We look forward to hearing from you regarding our submission and to respond to any further questions and comments you may have.

The authors’
